# VIRTUAL TAI CHI FOR OLDER VETERANS IMPACTED BY COVID-19

**DOI:** 10.1093/geroni/igad104.2317

**Published:** 2023-12-21

**Authors:** Bonnie Dawson, Hallie Keller, Linda Sawyer, Shannon Gorman, Jerome Sabangan, Adam McPartlin, Karl Brown, Dennis Sullivan

**Affiliations:** Central Arkansas Veterans Healthcare System, North Little Rock, Arkansas, United States; Central Arkansas VA Healthcare System, North Little Rock, Arkansas, United States; Central Arkansas Veterans Healthcare System, North Little Rock, Arkansas, United States; VA Palo Alto Health Care System, Palo Alto, California, United States; VA Palo Alto Healthcare System, Palo Alto, California, United States; Veteran’s Affairs Puget Sound, Seattle, Washington, United States; Puget Sound VA, Seattle, Washington, United States; Central Arkansas Veterans Healthcare System, North Little Rock, Arkansas, United States

## Abstract

As older Veterans (OV) were at risk for loneliness and deconditioning during the COVID pandemic, they were invited to join a Tai Chi program due to its known benefits in these areas. The objectives of this presentation are to examine outcomes, program adherence and feedback of OV enrolled in a 12-week Tai Chi course conducted virtually twice weekly at three VA sites during the COVID-19 pandemic. Participants completed the 30-Second Chair Stand (30CST) and the De Jong Gierveld Loneliness Scale (DJGS) at baseline and again when they finished the course. Attendance was recorded and end-course evaluations were also obtained. Univariate and multivariate statistics were used to summarize results. One hundred eleven OV aged 60 to 94 enrolled. Most were non-Hispanic (84%), white (72%), males (80%), living independently (92%) with a partner (43%). Of these, 86 (77.5%) completed a final assessment after attending a mean of 18.7 out of 24 sessions. Retention did not differ by site nor other demographics. Among the completers, there was not a significant change in DJGS (n=46, p=0.2748), but only 26.1% showed substantive loneliness at baseline. The 30CST improved significantly (n=72, p=0.0150) after controlling for site and baseline value. Most participants described the virtual course as rewarding, beneficial to their health and balance, and conducive to social connection. They also expressed desire to continue the program. Our results suggest OV achieve objective and subjective benefits from, and can feasibly participate in, a virtual Tai Chi program, which appears to be a valid alternative to in-person courses.

